# Spine Surgery in the Octogenarian Population: A Comparison of Demographics, Surgical Approach, and Healthcare Utilization With the PearlDiver Database

**DOI:** 10.7759/cureus.14561

**Published:** 2021-04-19

**Authors:** Chitra D Kumar, Nicholas Dietz, Mayur Sharma, Aurora Cruz, Christopher E Counts, Dengzhi Wang, Beatrice Ugiliweneza, Maxwell Boakye, Doniel Drazin

**Affiliations:** 1 Neurosurgery, University of Cincinnati College of Medicine, Cincinnati, USA; 2 Neurosurgery, University of Louisville School of Medicine, Louisville, USA; 3 Health Sciences, California State University Long Beach, Long Beach, USA; 4 Kentucky Spinal Cord Injury Research Center, University of Louisville, Louisville, USA; 5 Department of Health Management and Systems Science, University of Louisville, Louisville, USA; 6 Medicine, Pacific Northwest University of Health Sciences, Yakima, USA

**Keywords:** spine surgery, octogenarian, pearldiver, spinal stenosis, degenerative disease, decompression, fusion, healthcare utilization

## Abstract

Background

With the recent advances in technology and healthcare, increasing numbers of individuals over the age of 80 will require surgical intervention for spinal pathology. Given the risk of increased complications in the elderly, a limited number of spinal surgeries are performed on octogenarians every year. This makes it difficult to generalize the trends and outcomes of these surgeries to a greater population. This study attempts to understand the trends in the safety profile and healthcare utilization across the United States for octogenarians undergoing spinal fusion and/or decompression surgery for spinal stenosis and/or degenerative disease using the PearlDiver database.

Methodology

Patients who underwent fusion and/or decompression for stenosis and/or degenerative diseases were extracted using International Classification of Disease ninth and tenth revisions (ICD-9 prior to October 2015, ICD-10 after) from 2007 to 2016 in the PearlDiver database. Three comparative groups were considered: (1) primary fusion without concurrent decompression, (2) primary decompression with concurrent fusion, and (3) fusion with concurrent decompression. Outcomes of interest were patient characteristics, demographics, length of stay, surgery hospitalization payments, and discharge disposition. These outcomes were compared to patients over the age of 20 who also underwent spinal surgery.

Results

A total of 9,715 patients who underwent spinal surgery were identified in the search. Of the 9,139 patients, 503 were octogenarians and 73 were nonagenarians. Octogenarians and nonagenarians diagnosed with spinal stenosis were more likely to undergo decompression alone rather than fusion or both fusion and decompression (21 for both fusion and decompression; p < 0.0001). Patients diagnosed with both spinal stenosis and degeneration were more likely to undergo both fusion and decompression than fusion or decompression alone (239 for both, 208 for decompression alone, and 23 for fusion alone; p < 0.0001). No statistically significant difference was found in the percentage of patients discharged home following either fusion or decompression or both surgeries (p = 0.0737). The mean length of stay for patients in the 20-79-year age group was 2.79 days, whereas for the octogenarian and nonagenarian cohort it was 3.85 days. The index hospitalization pay for patients in the 20-79-year age group was $19,220, whereas for the octogenarians and nonagenarians cohort it was $15,091.

Conclusions

Patients over the age of 80 were more likely to undergo either a fusion procedure or a decompression procedure alone rather than both unless they were diagnosed with spinal degeneration. The PearlDiver database analysis indicates that the length of stay for octogenarians and nonagenarians is longer than that for patients in the 20-79-year age group, and that younger patients are more likely to be discharged earlier than patients over the age of 80. Moreover, we observed that the index hospitalization pay was higher for patients over the age of 20 than for octogenarians and nonagenarians in all cases except for a fusion procedure.

## Introduction

Considering recent advances in technology and healthcare, expected increases in longevity will necessitate alterations in the management of health systems. In the United States, the number of people age 65 and older is expected to double from 46 million in 2015 to over 98 million by the year 2060 [1]. Additionally, those aged 85 and older are anticipated to increase to 20 million by 2060, three times the current population for this age group. In an aging population, larger numbers of elderly individuals will require surgical intervention for spinal pathology [2]. Given the risk of increased complications in the elderly, the decision to perform surgery is based on weighing benefits and risks of the procedure in the context of patient comorbidities and preoperative disability [3]. Kalanithi and colleagues reported in 2009 that elderly individuals following posterior lumbar fusion were more likely to experience complications such as hematomas and pulmonary complications [4]. Additionally, Fineberg and colleagues observed in 2013 that elderly patients were more likely to experience postoperative delirium after lumbar spinal surgery which increased the length of stay in hospitals [5].

Those older than 65 have also been noted to incur higher hospitalization costs due to complications and comorbidities. For instance, Rollinghoff and colleagues noted in 2011 that patients over the age of 65 experienced a 50% failure from multilevel spinal fusions and had a 34.7% reoperation rate [6]. Moreover, a study that retrospectively analyzed 816 patients who underwent spine surgery between 2005 and 2008 found that there was an increase of $16,472 in hospital costs incurred by patients undergoing lumbar decompression and fusion between patients with the lowest and highest scores on the Charlson comorbidity index. This impact of comorbidities on the overall cost of spinal surgery was especially notable in older patients [7]. As the expected volume of required surgical interventions increases, information on the outcomes of spine surgery in an elderly population may assist surgeons on best practices and approaches to safely operate on older individuals [8].

To gain a better understanding of the trends in safety profile along with the healthcare utilization for octogenarians and nonagenarians compared to a younger population, we decided to utilize the PearlDiver database for our analysis. The PearlDiver database includes more than 4 billion patient records collected from analysis of private insurance claims from Humana and United Healthcare along with government claims from Medicare [8]. This repository has been used for research relating to spine surgery, opioid trends, mental illness, and knee arthroplasty. However, a few studies have used this database for research on spine surgery for octogenarians. From a limited sample of studies on older individuals in spine surgery, one reported on octogenarians receiving cervical fusion for spondylosis or lumbar fusion and was collected from 2005 to 2012 [9].

To address this perceived gap in the literature from PearlDiver on trends in spine surgery for older individuals, we analyzed the rates and healthcare utilization of fusion and decompression spine surgeries for spinal stenosis or degeneration between two groups: (1) patients who are 20-79 years old and (2) patients older than 80 years from 2007 to 2016. We anticipate that patients older than 80 years of age would be less likely to undergo both fusion and decompression surgeries together compared to those aged 20-79 years. Moreover, we expect that octogenarians and nonagenarians would have a longer length of stay post surgeries and higher healthcare utilization costs compared to nonagenarians.

## Materials and methods

Database

Data in this project include paid claims from Humana Inc extracted using PearlDiver patient record database. PearlDiver Technologies was located in Fort Wayne, IN, USA at the time of data extraction in 2019. Currently, it is located in Colorado Springs, CO, USA. PearlDiver is available to researchers for a fee.

Study cohort

Patients who underwent fusion and/or decompression for stenosis and/or degenerative diseases were extracted using International Classification of Disease ninth and tenth revisions (ICD-9 prior to October 2015, ICD-10 after) from 2007 to 2016. Three comparative groups were considered: (1) primary fusion without concurrent decompression, (2) primary decompression with concurrent fusion, and (3) either primary fusion with concurrent decompression or primary decompression with concurrent fusion. Patients who underwent concurrent kyphoplasty or vertebroplasty and those who were diagnosed with cancer were excluded. Only patients 20 years or older were included in the analysis.

Patient characteristics and outcomes

Outcomes of interest were length of stay, surgery hospitalization payments, and discharge disposition (home versus other). The following patients characteristics were noted at the time of surgery: age, sex, diagnosis type (primary stenosis with no concurrent degeneration, primary degeneration with no stenosis, and either primary stenosis with concurrent degeneration or primary degeneration with concurrent stenosis), geographic region (Midwest, Northeast, South, West), Charlson index [10] (0, 1, 2, 3, or more), and year of injury (2007-2016).

Statistical analysis

Characteristic and outcome variables were summarized with frequency count and percentages. The Chi-square test was used to compare different groups. All tests were two-sided, and results were concluded as statistically significant if the p-value was less than 0.05. All data preprocessing and statistical analyses were run using PearlDiver (Pearl-Diver Technologies, Fort Wayne, IN, USA).

## Results

Demographics for patients between 20 and 79 years of age

A total of 9,715 patients were identified in the search. Of the 9,139 patients older than 20 years, a greater number of patients between the ages of 20 and 79 years underwent surgery than those above the age of 80 (8,595 aged 20-79 versus 544 aged over 80; p < 0.0001) (Table 1). More females had both a fusion and decompression procedure than fusion or decompression alone (3,099 for both versus 1,312 for decompression and 455 for fusion, p < 0.0001). More patients above the age of 20 were diagnosed with both spinal stenosis and degeneration conditions than either spinal stenosis or degeneration (6,542 for both versus 1,002 for degeneration and 1,595 for spinal stenosis; p < 0.0001).

**Table 1:** Demographics for patients over 20 years of age.

Characteristics		All patients (n = 9,139)	Fusion alone (n = 844; 9.23%)	Decompression alone (n = 2,951; 32.3%)	Fusion and decompression (n = 5,344; 58.5%)	P-Value
Age at diagnosis						<0.0001>
	20-79, n (%)	8,595 (94.0)	807 (95.6)	2,696 (91.4)	5,092 (95.3)	
	Over 80, n (%)	544 (5.95)	37 (4.38)	255 (8.64)	252 (4.72)	
Gender, Female, n (%)		4,866 (53.2)	455 (53.9)	1,312 (44.5)	3,099 (58.0)	<0.0001>
Diagnosis type						<0.0001>
	Stenosis, n (%)	1,595 (17.5)	198 (23.5)	789 (26.7)	1,846 (34.5)	
	Degeneration, n (%)	1,002 (11.0)	229 (27.1)	316 (10.7)	608 (11.4)	
	Both, n (%)	6,542 (71.6)	417 (49.4)	1,846 (62.6)	457 (8.55)	
Regions						0.0199
	Midwest, n (%)	2,257 (24.7)	205 (24.3)	723 (24.5)	1,329 (24.9)	
	Northeast, n (%)	210 (2.30)	19 (2.25)	69 (2.34)	122 (2.28)	
	South, n (%)	5,567 (60.9)	551 (65.3)	1,794 (60.8)	3,222 (60.3)	
	West, n (%)	1,105 (12.1)	69 (8.17)	365 (12.4)	671 (12.6)	
Charlson index						<0.0001>
	0, n (%)	3,845 (42.1)	391 (46.3)	1,348 (45.7)	2,106 (39.4)	
	1, n (%)	2,226 (24.6)	188 (22.3)	695 (23.6)	1,343 (25.1)	
	2, n (%)	1,121 (12.3)	89 (10.5)	340 (11.5)	692 (12.9)	
	3+, n (%)	1,947 (21.3)	176 (20.9)	568 (19.3)	1,203 (22.5)	
Year						<0.0001>
	2007, n (%)	525 (5.74)	50 (5.92)	192 (6.51)	283 (5.30)	
	2008, n (%)	517 (5.66)	44 (5.21)	163 (5.52)	310 (5.80)	
	2009, n (%)	558 (6.10)	42 (4.98)	170 (5.76)	346 (6.47)	
	2010, n (%)	633 (6.93)	41 (4.86)	186 (6.30)	406 (7.60)	
	2011, n (%)	718 (7.86)	55 (6.52)	232 (7.86)	431 (8.07)	
	2012, n (%)	833 (9.11)	55 (6.52)	268 (9.08)	510 (9.54)	
	2013, n (%)	976 (10.7)	66 (7.82)	328 (11.1)	582 (10.9)	
	2014, n (%)	1,452 (15.9)	111 (13.2)	448 (15.2)	893 (16.7)	
	2015, n (%)	1,859 (20.3)	217 (25.7)	572 (19.4)	1,070 (20.0)	
	2016, n (%)	1,068 (11.7)	163 (19.3)	392 (13.3)	513 (9.60)	

More patients diagnosed with both spinal stenosis and degeneration underwent decompression alone rather than fusion or both decompression with fusion (1,846 for decompression versus 417 for fusion and 457 for both; p < 0.0001). Patients under the age of 80 diagnosed with stenosis were more likely to undergo both fusion and decompression (1,846 for both, 789 for decompression, and 198 for fusion; p < 0.0001). No statistically significant difference was found in surgery rates for patients under the age of 80 years among the different regions of the United States. Patients who scored a 0 on the Charlson index were more likely to undergo either decompression or fusion alone rather than both (46.3% for fusion, 45.7% for decompression, and 39.4% for both; p < 0.0001). Patients with higher scores on the Charlson index were more likely to have both fusion and decompression than either by itself (p < 0.0001). From 2007 to 2016, more patients had both a fusion and decompression procedure than either by itself. The data also display a trend of increasing number of patients undergoing surgery for spinal stenosis and/or degeneration from 2007 to 2015, but in 2016 there was a drop in the number of patients who underwent surgery (from 1,859 in 2015 to 1,068 in 2016).

Demographics for patients 80 years of age or older

The dataset contained 576 patients over the age of 80 (Table 2). Of these patients, 503 were octogenarians and 73 were nonagenarians. No statistically significant difference was found in the kind of surgery utilized for octogenarians and nonagenarians (p = 0.3409). Patient gender was also not statistically significant among the surgeries (p = 0.2478). Patients in this age group were more likely to be diagnosed with both spinal stenosis and degeneration than either alone (81.6% for both, 4.69% for degeneration, and 13.7% for spinal stenosis; p < 0.0001). Octogenarians and nonagenarians diagnosed with spinal stenosis were more likely to undergo decompression alone rather than fusion or both fusion and decompression (21 for both fusion and decompression; p < 0.0001). Patients diagnosed with both spinal stenosis and degeneration were more likely to undergo both fusion and decompression than fusion or decompression alone (239 for both, 208 for decompression alone, and 23 for fusion alone; p < 0.0001). No difference was found in surgery rates among the different regions of the United States (p = 0.5463) or the score patients had on the Charlson index (p = 0.54172). Moreover, there was no difference in the rate of different surgeries from 2007 to 2016 (p = 0.7765).

**Table 2:** Demographics for patients over 80 years old. *sample size <11 masked to preserve patients’ privacy; ** sample size masked to avoid the deduction of the sample size that is <11

Characteristic		All patients (n = 576; 100%)	Fusion alone (n = 39; 6.77%)	Decompression alone (n = 265; 46.0%)	Fusion and decompression (n = 272; 47.2%)	P-Value
Age at diagnosis						0.3409
	80-89, n (%)	503 (87.3)	**	**	236 (86.8)	
	Over 90, n (%)	73 (12.7)	*	**	36 (13.2)	
Gender, Female, n (%)		362 (62.8)	25 (64.1)	157 (59.2)	180 (66.2)	0.2478
Diagnosis type						<0.0001>
	Stenosis, n (%)	79 (13.7)	*	**	21 (7.72)	
	Degeneration, n (%)	27 (4.69)	*	*	12 (4.41)	
	Both, n (%)	470 (81.6)	23 (59.0)	208 (78.5)	239 (87.7)	
Regions						0.5463
	Midwest, n (%)	166 (28.8)	10 (25.6)	71 (26.8)	85 (31.3)	
	Northeast, n (%)	20 (3.47)	*	**	7 (2.57)	
	South, n (%)	300 (52.1)	21 (53.8)	146 (55.1)	133 (48.9)	
	West, n (%)	90 (15.6)	*	**	47 (17.3)	
Charlson index						0.5172
	0, n (%)	150 (26.0)	*	**	69 (25.4)	
	1, n (%)	99 (17.2)	*	**	48 (17.6)	
	2, n (%)	97 (16.8)	*	**	41 (15.1)	
	3+, n (%)	230 (29.9)	19 (48.7)	97 (36.6)	114 (41.9)	
Year						0.7765
	2007, n (%)	33 (5.73)	*	**	15 (5.51)	
	2008, n (%)	37 (6.42)	*	**	19 (6.98)	
	2009, n (%)	30 (5.21)	*	**	16 (5.88)	
	2010, n (%)	57 (9.89)	*	**	22 (8.09)	
	2011, n (%)	63 (10.9)	*	**	28 (10.3)	
	2012, n (%)	47 (8.16)	*	**	29 (10.7)	
	2013, n (%)	81 (14.1)	*	**	39 (14.3)	
	2014, n (%)	82 (14.2)	*	**	43 (15.8)	
	2015, n (%)	101 (17.5)	*	**	44 (16.2)	
	2016, n (%)	45 (7.81)	*	**	17 (6.25)	

Healthcare utilization for patients 20-79 years of age

The mean length of stay for all patients was 2.79 days (SD = 4.44) (Table 3). For patients who underwent fusion alone, the mean length of stay was 3.01 days; for those who had decompression alone, the mean length of stay was 0.81 days; and for those who had both decompression and fusion, the mean length of stay was 3.83 days (p < 0.0001). The mean index hospitalization pay for patients who underwent fusion alone was $20,999, for decompression alone was $4,052, and for both fusion and decompression was $21,127 (p < 0.0001). Overall, 45% of the patients who had only the fusion procedure done were discharged home, whereas of those who had only the decompression procedure done, 31% were discharged home. Of those who had both the fusion and decompression procedures, 64.4% were discharged home (p < 0.0001).

**Table 3:** Unadjusted comparison of outcomes for patients over 20 years old.

	All patients	Fusion alone	Decompression alone	Fusion and decompression	P-Value
n (%)	9,139 (100%)	844 (9.23%)	2,951 (32.3%)	5,344 (58.5%)	
Length of stay, mean (SD)	2.79 (4.44)	3.01 (4.94)	0.81 (4.64)	3.83 (4.34)	<0.0001>
Index hospitalization pay, mean (SD)	19,220 (19,324)	20,999 (23,715)	4,052 (4,799)	27,127 (21,821)	<0.0001>
Discharge home, n (%)	4,745 (51.9)	386 (45.7)	916 (31.0)	3,443 (64.4)	<0.0001>

Healthcare utilization for patients 80 years of age or older

The mean length of stay for octogenarians and nonagenarians was 6.33 days after fusion alone, 1.39 days after decompression alone, and 5.69 days after both fusion and decompression (p < 0.0001) (Table 4). The mean index hospitalization pay for those who underwent fusion alone was $22,730. For those who only had the decompression procedure, the mean index hospitalization pay was $3,896, and for those who underwent both fusion and decompression, the mean was $24,267 (p < 0.0001). No statistically significant difference was found in the percentage of patients discharged home following either fusion or decompression or both surgeries (p = 0.0737).

**Table 4:** Unadjusted comparison of outcomes for patients 80+ years old.

	All patients (n = 576; 100%)	Fusion alone (n = 39; 6.77%)	Decompression alone (n = 265; 46.0%)	Fusion and decompression (n = 272; 47.2%)	P-Value
Length of stay, mean (SD)	3.85 (6.64)	6.33 (6.87)	1.39 (6.97)	5.69 (6.48)	<0.0001>
Index hospitalization pay, mean (SD)	15,091 (13,900)	22,730 (19,650)	3,896 (5,339)	24,267 (15,868)	<0.0001>
Discharge home, n (%)	193 (33.5)	12 (30.8)	77 (29.1)	104 (38.2)	0.0737

Database comparison

Using previously published data, a comparison for diagnosis, procedure, and outcomes in octogenarians and nonagenarians was conducted among the PearlDiver, MarketScan, National Inpatient Sample (NIS), and National Surgical Quality Improvement Program (NSQIP) databases [11] (Table 5). MarketScan, NIS, and NSQIP showed that patients were more likely to be diagnosed with spinal stenosis than degeneration (MarketScan: 79.55% patients were diagnosed with stenosis versus 20.44% with degeneration; NIS: 80.29% patients were diagnosed with stenosis versus 19.71% with degeneration, and NSQIP: 84.13% patients were diagnosed with stenosis versus 15.87% with degeneration), whereas the PearlDiver database showed that patients were more likely to be diagnosed with both spinal stenosis and degeneration (81.6% patients were diagnosed with both stenosis and degeneration versus 13.7% with only spinal stenosis and 4.69% with only degeneration). MarketScan and NIS data present that patients were equally likely to have undergone either fusion or decompression (MarketScan: 49.80% for fusion and 50.19% for decompression; NIS: 48.41% for fusion and 51.59% for decompression). According to NSQIP data, patients over the age of 80 diagnosed with either spinal stenosis or degeneration were more likely to undergo a decompression procedure (67.74%) than a fusion procedure. However, the PearlDiver data show that 6.4% of patients had a fusion procedure, 46% had a decompression procedure, and around 47% had both fusion and decompression. All four databases showed approximately the same length of hospitalization stay for surgery for spinal stenosis or degeneration (PearlDiver: 3.85, MarketScan: 3.6, NIS: 3.8, and NSQIP: 3.9).

**Table 5:** Comparison of primary diagnoses, procedures, and length of stay for different databases.

	PearlDiver (n = 576)	MarketScan (n = 15,105)	National Inpatient Sample (n = 40,854)	National Surgical Quality Improvement Program (n = 7,682)
Gender, Female, n (%)	362 (62.8%)	7,974 (52.79%)	22,224 (54.3%)	3,835 (49.93%)
Comorbidities				
0, n (%)	150 (26.0)	5,551 (36.75)	7,842 (19.2)	788 (10.26)
1, n (%)	99 (17.2)	6,141 (40.66)	18,479 (45.23)	2,474 (32.21)
2, n (%)	97 (16.8)	2,764 (18.3)	10,838 (26.53)	2,668 (34.73)
3+, n (%)	230 (29.9)	649 (4.3)	3,695 (9.04)	1,752 (22.81)
Diagnosis type				
Spinal stenosis, n (%)	79 (13.7)	7,913 (79.55)	21,129 (80.29)	4,256 (84.13)
Degeneration, n (%)	27 (4.69)	2,033 (20.44)	5,188 (19.71)	803 (15.87)
Both, n (%)	470 (81.6)	N/A	N/A	N/A
Procedures, n (%)				
Fusion	39 (6.4)	4,954 (49.80)	12,739 (48.41)	1,632 (32.26)
Decompression	265 (46.0)	4,992 (50.19)	13,578 (51.59)	3,427 (67.74)
Both	272 (47.22)	N/A	N/A	N/A
Length of stay, mean (SD)	3.85 (6.64)	3.6 (3.5)	3.8 (3.5)	3.5 (3.9)

## Discussion

National databases, such as PearlDiver, allow us to appreciate the trends and healthcare utilization for neurosurgeries in an aging population. Our results indicate that the length of stay for octogenarians is longer than that for patients over 20 years old, and that younger patients are more likely to be discharged home than patients over the age of 80. Surprisingly, in contrast to our hypothesis, the results show that the index hospitalization pay was higher for patients over the age of 20 than for octogenarians in all cases except for a fusion procedure. Furthermore, it is interesting to note the types of surgeries different populations had for different conditions. For example, patients over the age of 80 were more likely to undergo either a fusion procedure or a decompression procedure alone unless they were diagnosed with degeneration (in which case they were more likely to have a fusion and a decompression surgery).

Carreon et al. conducted a retrospective cohort review published in 2003 using the hospital records of 98 patients who were 65 years of age or older when they underwent surgery for degenerative disc diseases [12]. Results showed that perioperative complications occurred in 78 of the patients, and the study concluded that elderly patients were more at risk for surgical complications due to their age, which can help us understand the longer length of stay observed with older individuals in this study.

Given that the rate of complication increases with the addition of a fusion procedure, patients over the age of 80 are more likely to undergo decompression alone to decrease the risk of more complex surgical procedures with longer duration [13-17]. Transfeldt et al. showed that in the context of degenerative scoliosis with radiculopathy, the complication rate for decompression alone in patients averaging 76.4 years of age was 10% [18]. On the other hand, for patients averaging 70.4 years of age who underwent both a decompression and a fusion procedure, the complication rate increased to 40% [18].

Still, patients diagnosed with degeneration were more likely to undergo both fusion and decompression regardless of age. Yavin et al. conducted a systematic review that showed the risk of reoperation following decompression and fusion for degenerative spondylolisthesis was lower than the risk of reoperation with decompression alone [19]. Yagi et al. presented a similar conclusion after conducting a retrospective review of 99 patients with a three-year follow-up [20].

Liang et al. performed a systematic review along with a meta-analysis of the data collected in 2017 to show that combined decompression and fusion for degenerative spondylolisthesis led to greater improvement in clinical satisfaction and reduction of postoperative leg pain compared to decompression alone [21]. Interestingly, some studies also show the opposite to be true, stating that decompression alone is enough for degenerative conditions without the need for an additional fusion [22,23].

We showed that patients aged 20-79 who were diagnosed with both spinal stenosis and degenerative conditions were more likely to undergo decompression alone, whereas those over the age 80 underwent both fusion and decompression. Deyo et al. conducted a retrospective cohort analysis of Medicare claims from 2002 to 2007 in order to analyze complications rates and trends associated with surgery in older patients for spinal stenosis [16]. They showed that from 2002, the frequency of fusion procedures increased for spinal stenosis. Kepler et al. also conducted a retrospective review from 1999 to 2011 of patients who underwent surgery for degenerative spondylolisthesis and demonstrated that rates of fusion increased substantially compared to rates of decompression alone [24]. This data might help explain why patients are undergoing both fusion and decompression rather than decompression alone. Evidence supports that while there is a decline in patients receiving decompression for spinal stenosis over the age of 80, those receiving surgery over the age of 90 have not shown higher rates of reoperation or compaction than those aged 80-89 [25]. Ahmad et al. conducted a prospective cohort study with 83 patients who had both spinal stenosis and degenerative spondylolisthesis to understand if fusion was necessary [26]. Fusion was not necessary to improve back and leg pain.

The use of databases for understanding trends in hospitalization utilization and surgery trends has increased over the years. Therefore, it is important to understand how these trends compare among databases [27]. This is the first study bringing together data on demographics of elderly patients undergoing fusion or decompression from four commonly used databases. We observed that MarketScan, NIS, and NSQIP had a much greater sample size than PearlDiver for patients over the age of 80 undergoing surgery for spinal stenosis or degenerative conditions. This could be because the PearlDiver database captures only about 13% of the US population with private insurance, whereas the NIS is the largest database on inpatient stays and MarketScan involves information from commercial insurance and primary care electronic records along with Medicare and Medicaid supplemental databases [28].

Rates of fusion and decompression were similar in both NIS and MarketScan databases. However, according to the PearlDiver database, rates of decompression alone or decompression along with fusion were greater than fusion alone. NSQIP had greater rates of decompression than fusion. This could be because NSQIP patients are less healthy than the patients from the MarketScan database making fusion more difficult [11]. All four databases show approximately the same length of stay for elderly patients undergoing spine surgery.

Strengths and limitations

This study has several limitations. We understand that degenerative disc disease can lead to spinal stenosis, and the data presented in the tables might make them appear as two different entities. This is mainly due to the manner in which data are recorded in these databases. We do realize it is important to keep this in consideration while analyzing the data presented. We also realize that it is important to keep in mind that the population size for the 20-79-year-old cohort is much greater than that for the octogenarian and nonagenarian cohort, which can introduce bias to the analysis.

Future studies may obtain data on fusion and decompression rates for NIS, NSQIP, and MarketScan to better understand the trend of surgeries for degenerative disease and spinal stenosis. Moreover, it is important to consider the differences in demographics and data collection for the four databases before we begin to generalize these results to the US population. A notable limitation in utilizing national databases is that they are dependent on ICD-9 or CPT coding to collect data on diagnoses, procedures, and complications. Due to clerical error or different interpretations, there could be a discrepancy in the ICD-9 or CPT codes logged which would then affect the trend pattern observed during research [8]. We also cannot match patients among the different databases which limits our ability to deduce different outcomes.

MarketScan, NSQIP, and PearlDiver include longitudinal tracking and follow patient outcomes after the perioperative period. On the other hand, NIS does not and only collects data while the patient is admitted in the hospital. Among the MarketScan, NSQIP, and PearlDiver databases, NSQIP only follows patient outcomes for 30 days post-discharge, thereby limiting the conclusions that can be drawn regarding the long-term efficacy of certain surgeries with certain demographics. Another potential limitation includes the sampling strategies used across different national databases, especially as a database can choose to use a new sampling strategy as NIS did in 2012 [8]. This can complicate any temporal trends observed in these studies.

A meta-analysis has shown that statistically significant study results can change depending on the kind of database utilized [29]. Therefore, it is important to keep in mind that the statistical significance shown in one study does not directly correlate to clinical significance, and that there is a limit to the generalizability of the comparison of results and even the comparison across different databases.

## Conclusions

The PearlDiver database analysis indicates that the length of stay for octogenarians is longer than that for patients over 20 years old, and that younger patients are more likely to be discharged home than patients over the age of 80. Moreover, we observed that the index hospitalization pay was higher for patients over the age of 20 than for octogenarians in all cases except for a fusion procedure. Patients over the age of 80 were more likely to undergo either a fusion procedure or a decompression procedure alone rather than both unless they were diagnosed with spinal degeneration.

## Appendices

**Table 6:** Summary of ICD-9/10 codes utilized to query data from the PearlDiver database ICD-9/10, International Classification of Disease ninth/tenth revisions; CPT-4, Current Procedural Terminology, 4th Editio

	ICD-9	ICD-10	CPT-4
Diagnoses			
Stenosis	72,400, 72,401, 72,402	M4800 - M4808, M48.061	NA
Degeneration	7,241, 7,243, 72,433, 7,245, 7,249, 73,611, 7,384, 75,611, 75,612, 75,619	M546, M5430, M5489, M549, M438X9, M539, M4300, M4310, Q762, Q506	NA
Cancer	140-239	C00, C000:C009, C01, C02, C020:C029, C03, C030:C0039, C04, C040:C049, C05, C050:C059, C06, C060:C060:C069, C07, C070, C08, C080:C089, C09, C091, C099, C10, C100:C109, C11, C111:C119, C12, C120:C13, C130:C139, C14, C140:C148	NA
Procedures			
Fusion	8,100	0RG0070, 0RG0071, 0RG007J, 0RG00J0, 0RG00J1, 0RG00JJ, 0RG00K0, 0RG00K1, 0RG00KJ, 0RG00Z0, 0RG00Z1, 0RG00ZJ, 0RG0370, 0RG0371, 0RG037J, 0RG03J0, 0RG03J1, 0RG03JJ, 0RG03K0, 0RG03K1, 0RG03KJ, 0RG03Z0, 0RG03Z1, 0RG03ZJ, 0RG0470, 0RG0471, 0RG047J, 0RG04J0, 0RG04J1, 0RG04JJ, 0RG04K0, 0RG04K1, 0RG04KJ, 0RG04Z0, 0RG04Z1, 0RG04ZJ, 0RG1070, 0RG1071, 0RG107J, 0RG10J0, 0RG10J1, 0RG10JJ, 0RG10K0, 0RG10K1, 0RG10KJ, 0RG10Z0, 0RG10Z1, 0RG10ZJ, 0RG1370, 0RG1371, 0RG137J, 0RG13J0, 0RG13J1, 0RG13JJ, 0RG13K0, 0RG13K1, 0RG13KJ, 0RG13Z0, 0RG13Z1, 0RG13ZJ, 0RG1470, 0RG1471, 0RG147J, 0RG14J0, 0RG14J1, 0RG14JJ, 0RG14K0, 0RG14K1, 0RG14KJ, 0RG14Z0, 0RG14Z1, 0RG14ZJ, 0RG4070, 0RG4071, 0RG407J, 0RG40J0, 0RG40J1, 0RG40JJ, 0RG40K0, 0RG40K1, 0RG40KJ, 0RG40Z0, 0RG40Z1, 0RG40ZJ, 0RG4370, 0RG4371, 0RG437J, 0RG43J0, 0RG43J1, 0RG43JJ, 0RG43K0, 0RG43K1, 0RG43KJ, 0RG43Z0, 0RG43Z1, 0RG43ZJ, 0RG4470, 0RG4471, 0RG447J, 0RG44J0, 0RG44J1, 0RG44JJ, 0RG44K0, 0RG44K1, 0RG44KJ, 0RG44Z0, 0RG44Z1, 0RG44ZJ, 0RG6070, 0RG6071, 0RG607J, 0RG60J0, 0RG60J1, 0RG60JJ, 0RG60K0, 0RG60K1, 0RG60KJ, 0RG60Z0, 0RG60Z1, 0RG60ZJ, 0RG6370, 0RG6371, 0RG637J, 0RG63J1, 0RG63JJ, 0RG63K0, 0RG63K1, 0RG63KJ, 0RG63Z0, 0RG63Z1, 0RG63ZJ, 0RG6470, 0RG6471, 0RG647J, 0RG64J0, 0RG64J1, 0RG64JJ, 0RG64K0, 0RG64K1, 0RG64KJ, 0RG64Z0, 0RG64Z1, 0RG64ZJ, 0RGA070, 0RGA071, 0RGA07J, 0RGA0J0, 0RGA0J1, 0RGA0JJ, 0RGA0K0, 0RGA0K1, 0RGA0KJ, 0RGA0Z0, 0RGA0Z1, 0RGA0ZJ, 0RGA370, 0RGA371, 0RGA37J, 0RGA3J0, 0RGA3J1, 0RGA3JJ, 0RGA3K0, 0RGA3K1, 0RGA3KJ, 0RGA3Z0, 0RGA3Z1, 0RGA3ZJ, 0RGA470, 0RGA471, 0RGA47J, 0RGA4J0, 0RGA4J1, 0RGA4JJ, 0RGA4K0, 0RGA4K1, 0RGA4KJ, 0RGA4Z0, 0RGA4Z1, 0RGA4ZJ, 0SG0070, 0SG0071, 0SG007J, 0SG00J0, 0SG00J1, 0SG00JJ, 0SG00K0, 0SG00K1, 0SG00KJ, 0SG00Z0, 0SG00Z1, 0SG00ZJ, 0SG0370, 0SG0371, 0SG037J, 0SG03J0, 0SG03J1, 0SG03JJ, 0SG03K0, 0SG03K1, 0SG03KJ, 0SG03Z0, 0SG03Z1, 0SG03ZJ, 0SG0470, 0SG0471, 0SG047J, 0SG04J0, 0SG04J1, 0SG04JJ, 0SG04K0, 0SG04K1, 0SG04KJ, 0SG04Z0, 0SG04Z1, 0SG04ZJ, 0SG3070, 0SG3071, 0SG307J, 0SG30J0, 0SG30J1, 0SG30JJ, 0SG30K0, 0SG30K1, 0SG30KJ, 0SG30Z0, 0SG30Z1, 0SG30ZJ, 0SG3370, 0SG3371, 0SG337J, 0SG33J0, 0SG33J1, 0SG33JJ, 0SG33K0, 0SG33K1, 0SG33KJ, 0SG33Z0, 0SG33Z1, 0SG33ZJ, 0SG3470, 0SG3471, 0SG347J, 0SG34J0, 0SG34J1, 0SG34JJ, 0SG34K0, 0SG34K1, 0SG34KJ, 0SG34Z0, 0SG34Z1, 0SG34ZJ, 0SG504Z, 0SG507Z, 0SG50JZ, 0SG50KZ, 0SG50ZZ, 0SG534Z, 0SG537Z, 0SG53JZ, 0SG53KZ, 0SG53ZZ, 0SG544Z, 0SG547Z, 0SG54JZ, 0SG54KZ, 0SG54ZZ, 0SG604Z, 0SG607Z, 0SG60JZ, 0SG60KZ, 0SG60ZZ, 0SG634Z, 0SG637Z, 0SG63JZ, 0SG63KZ, 0SG63ZZ, 0SG644Z, 0SG647Z, 0SG64JZ, 0SG64KZ, 0SG64ZZ, 8101, 0RG0070, 0RG0071 , 0RG007J, 0RG00J0, 0RG00J1, 0RG00JJ, 0RG00K0, 0RG00K1, 0RG00KJ, 0RG00Z0, 0RG00Z1, 0RG00ZJ, 0RG0370, 0RG0371, 0RG037J, 0RG03J0, 0RG03J1, 0RG03JJ, 0RG03K0, 0RG03K1, 0RG03KJ, 0RG03Z0, 0RG03ZJ, 0RG0470, 0RG0471, 0RG047J, 0RG04J0, 0RG04J1, 0RG04JJ, 0RG04K0, 0RG04K1, 0RG04KJ, 0RG04Z0, 0RG04Z1, 0RG04ZJ, 8102, 0RG1070, 0RG10J0, 0RG10K0, 0RG10Z0, 0RG1370, 0RG13J0, 0RG13K0, 0RG13Z0, 0RG1470, 0RG14J0, 0RG14K0, 0RG14Z0, 0RG4070, 0RG40J0, 0RG40K0, 0RG40Z0, 0RG4370, 0RG43J0, 0RG43K0, 0RG43Z0, 0RG4470, 0RG44J0, 0RG44K0, 0RG44Z0, 8103, 0RG1071, 0RG10J1, 0RG10K1, 0RG10Z1, 0RG1371, 0RG13J1, 0RG13K1, 0RG13Z1, 0RG1471, 0RG14J1, 0RG14K1, 0RG14Z1, 0RG4071, 0RG40J1, 0RG40K1, 0RG40Z1, 0RG4371, 0RG43J1, 0RG43K1, 0RG43Z1, 0RG4471, 0RG44J1, 0RG44K1, 0RG44Z1, 8104, 0RG6070, 0RG60J0, 0RG60K0, 0RG60Z0, 0RG6370, 0RG63J0, 0RG63K0, 0RG63Z0, 0RG6470, 0RG64J0, 0RG64K0, 0RG64Z0, 0RGA070, 0RGA0J0, 0RGA0K0, 0RGA0Z0, 0RGA370, 0RGA3J0, 0RGA3K0, 0RGA3Z0, 0RGA470, 0RGA4J0, 0RGA4K0, 0RGA4Z0, 8105, 0RG6071, 0RG60J1, 0RG60K1, 0RG60Z1, 0RG6371, 0RG63J1, 0RG63K1, 0RG63Z1, 0RG6471, 0RG64J1, 0RG64K1, 0RG64Z1, 0RGA071, 0RGA0J1, 0RGA0K1, 0RGA0Z1, 0RGA371, 0RGA3J1, 0RGA3K1, 0RGA3Z1, 0RGA471, 0RGA4J1, 0RGA4K1, 0RGA4Z1, 8106, 0SG0070, 0SG00J0, 0SG00K0, 0SG00Z0, 0SG0370, 0SG03J0, 0SG03K0, 0SG03Z0, 0SG0470, 0SG04J0, 0SG04K0, 0SG04Z0, 0SG3070, 0SG30J0, 0SG30K0, 0SG30Z0, 0SG3370, 0SG33J0, 0SG33K0, 0SG33Z0, 0SG3470, 0SG34J0, 0SG34K0, 0SG34Z0, 8107, 0SG0071, 0SG00J1, 0SG00K1, 0SG00Z1, 0SG0371, 0SG03J1, 0SG03K1, 0SG03Z1, 0SG0471, 0SG04J1, 0SG04K1, 0SG04Z1, 0SG3071, 0SG30J1, 0SG30K1, 0SG30Z1, 0SG3371, 0SG33J1, 0SG33K1, 0SG33Z1, 0SG3471, 0SG34J1, 0SG34K1, 0SG34Z1, 8108, 0SG007J, 0SG00JJ, 0SG00KJ, 0SG00ZJ, 0SG037J, 0SG03JJ, 0SG03KJ, 0SG03ZJ, 0SG047J, 0SG04JJ, 0SG04KJ, 0SG04ZJ, 0SG307J, 0SG30JJ, 0SG30KJ, 0SG30ZJ, 0SG337J, 0SG33JJ, 0SG33KJ, 0SG33ZJ, 0SG347J, 0SG34JJ, 0SG34KJ, 0SG34ZJ, 0SG704Z, 0SG707Z, 0SG70JZ, 0SG70KZ, 0SG70ZZ, 0SG734Z, 0SG737Z, 0SG73JZ, 0SG73KZ, 0SG73ZZ, 0SG744Z, 0SG747Z, 0SG74JZ, 0SG74KZ, 0SG74ZZ, 0SG804Z, 0SG807Z, 0SG80JZ, 0SG80KZ, 0SG80ZZ, 0SG834Z, 0SG837Z, 0SG83JZ, 0SG83KZ, 0SG83ZZ, 0SG844Z, 0SG847Z, 0SG84JZ, 0SG84KZ, 0SG84ZZ	22,548, 22,551, 22,552, 22,554, 22,556, 22,558, 22,586, 22,590, 22,595, 22,600, 22,614, 22,630, 22,632, 22,633, 22,634, 22,641, 22,800, 22,802, 22,804, 22,808, 22,810, 22,812
Decompression	0301	00C30ZZ, 00C33ZZ, 00C34ZZ, 00CT0ZZ, 00CT3ZZ, 00CT4ZZ, 00CU0ZZ, 00CU3ZZ, 00CU4ZZ, 00CW0ZZ, 00CW3ZZ, 00CW4ZZ, 00CX0ZZ, 00CX3ZZ, 00CX4ZZ, 00CY0ZZ, 00CY3ZZ, 00CY4ZZ, 8050, 0R530ZZ, 0R533ZZ, 0R534ZZ, 0R550ZZ, 0R553ZZ, 0R554ZZ, 0R590ZZ, 0R593ZZ, 0R594ZZ, 0R5B0ZZ, 0R5B3ZZ, 0R5B4ZZ, 0RB30ZZ, 0RB33ZZ, 0RB34ZZ, 0RB50ZZ, 0RB53ZZ, 0RB54ZZ, 0RB90ZZ, 0RB93ZZ, 0RB94ZZ, 0RBB0ZZ, 0RBB3ZZ, 0RBB4ZZ, 0RT30ZZ, 0RT40ZZ, 0RT50ZZ, 0RT90ZZ, 0RTB0ZZ, 0S520ZZ, 0S523ZZ, 0S524ZZ, 0S540ZZ, 0S543ZZ, 0S544ZZ, 0SB20ZZ, 0SB23ZZ, 0SB24ZZ, 0SB40ZZ, 0SB43ZZ, 0SB44ZZ, 0ST20ZZ, 0ST40ZZ, 8051, 0RB30ZZ, 0RB33ZZ, 0RB34ZZ, 0RB50ZZ, 0RB53ZZ, 0RB54ZZ, 0RB90ZZ, 0RB93ZZ, 0RB94ZZ, 0RBB0ZZ, 0RBB3ZZ, 0RBB4ZZ, 0RT30ZZ, 0RT40ZZ, 0RT50ZZ, 0RT90ZZ, 0RTB0ZZ, 0SB20ZZ, 0SB23ZZ, 0SB24ZZ, 0SB40ZZ, 0SB43ZZ, 0SB44ZZ, 0ST20ZZ, 0ST40ZZ, 7849, 0PN30ZZ, 0PN33ZZ, 0PN34ZZ, 0PN40ZZ, 0PN43ZZ, 0PN44ZZ, 0PNT0ZZ, 0PNT3ZZ, 0PNT4ZZ, 0PNV0ZZ, 0PNV3ZZ, 0PNV4ZZ, 0PQ30ZZ, 0PQ33ZZ, 0PQ34ZZ, 0PQ3XZZ, 0PQ40ZZ, 0PQ43ZZ, 0PQ44ZZ, 0PQ4XZZ, 0PQT0ZZ, 0PQT3ZZ, 0PQT4ZZ, 0PQTXZZ, 0PQV0ZZ, 0PQV3ZZ, 0PQV4ZZ, 0PQVXZZ, 0PU30JZ, 0PU40JZ, 0PUT0JZ, 0PUT3JZ, 0PUT4JZ, 0PUV0JZ, 0PUV3JZ, 0PUV4JZ, 0QN00ZZ, 0QN03ZZ, 0QN04ZZ, 0QN20ZZ, 0QN23ZZ, 0QN24ZZ, 0QN30ZZ, 0QN33ZZ, 0QN34ZZ, 0QNQ0ZZ, 0QNQ3ZZ, 0QNQ4ZZ, 0QNR0ZZ, 0QNR3ZZ, 0QNR4ZZ, 0QQ00ZZ, 0QQ03ZZ, 0QQ04ZZ, 0QQ0XZZ, 0QQ20ZZ, 0QQ23ZZ, 0QQ24ZZ, 0QQ2XZZ, 0QQ30ZZ, 0QQ33ZZ, 0QQ34ZZ, 0QQ3XZZ, 0QQD3ZZ, 0QQF3ZZ, 0QQQ0ZZ, 0QQQ3ZZ, 0QQQ4ZZ, 0QQQXZZ, 0QQR0ZZ, 0QQR3ZZ, 0QQR4ZZ, 0QQRXZZ, 0QU00JZ, 0QU20JZ, 0QU23JZ, 0QU24JZ, 0QU30JZ, 0QU33JZ, 0QU34JZ, 0QUQ0JZ, 0QUQ3JZ, 0QUQ4JZ, 0QUR0JZ, 0QUR3JZ, 0QUR4JZ	63,001, 63,003, 63,005, 63,011, 63,012, 63,013, 63,015, 63,016, 63,017, 63,020, 63,030, 63,040, 63,041, 63,042, 63,045, 63,046, 63,047, 63,055, 63,056, 63,057
Kyphoplasty	8,166	0PS33ZZ, 0PU33JZ, 0PS43ZZ, 0PU43JZ, 0QS03ZZ, 0QU03JZ, 0QS13ZZ, 0QU13JZ, 0QSS3ZZ, 0QUS3JZ	22,525
Vertebroplasty	8,165	0PU33JZ, 0PU34JZ, 0PU43JZ, 0PU44JZ, 0QU03JZ, 0QU04JZ, 0QU13JZ, 0QU14JZ	22,522
